# Regulatory roles of tRNA-derived RNA fragments in human pathophysiology

**DOI:** 10.1016/j.omtn.2021.06.023

**Published:** 2021-07-02

**Authors:** Kush Kumar Pandey, Deeksha Madhry, Y.S. Ravi Kumar, Shivani Malvankar, Leena Sapra, Rupesh K. Srivastava, Sankar Bhattacharyya, Bhupendra Verma

**Affiliations:** 1 Department of Biotechnology, All India Institute of Medical Sciences, Ansari Nagar, New Delhi 110029, India; 2 Department of Biotechnology, M.S. Ramaiah, Institute of Technology, MSR Nagar, Bengaluru, India; 3 Translational Health Science and Technology Institute, NCR Biotech Science Cluster, Faridabad, India

**Keywords:** sncRNA, lncRNA, tRFs, tsRNA, tiRNA, tRNA processing, RNases, stress, pathophysiology, human diseases

## Abstract

Hundreds of tRNA genes and pseudogenes are encoded by the human genome. tRNAs are the second most abundant type of RNA in the cell. Advancement in deep-sequencing technologies have revealed the presence of abundant expression of functional tRNA-derived RNA fragments (tRFs). They are either generated from precursor (pre-)tRNA or mature tRNA. They have been found to play crucial regulatory roles during different pathological conditions. Herein, we briefly summarize the discovery and recent advances in deciphering the regulatory role played by tRFs in the pathophysiology of different human diseases.

## Graphical abstract

**Figure:**
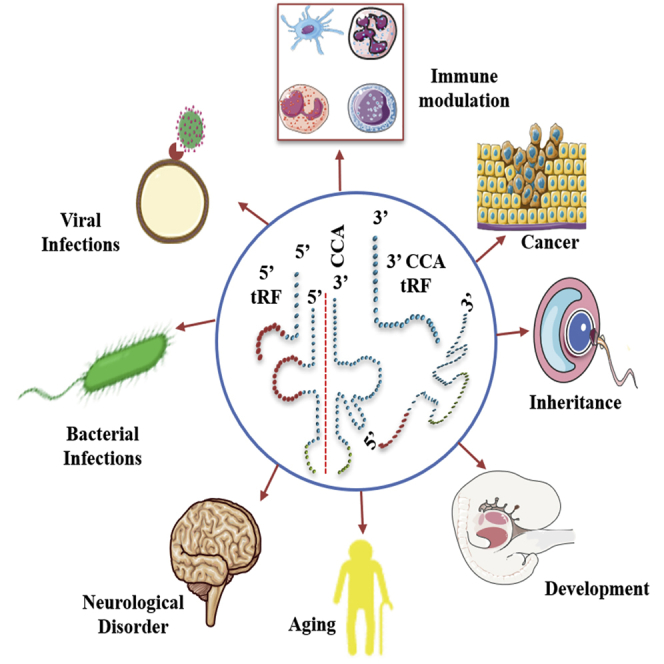

## Teaser

Insights into the potential of RNA therapeutics have led to the extensive research progress in the arena of non-coding RNAs. This review provides insight about the tRNA-derived RNA fragments and summarizes their various functional implications in the pathophysiology of various human diseases.

## Introduction

The human genome transcribes coding as well as non-coding transcripts. Coding transcripts are mainly mRNAs that are translated into proteins. Non-coding transcripts do not code for any protein, rather they play a significant role in the regulation of most protein-coding genes. Non-coding transcripts primarily comprise rRNAs, tRNAs, small nucleolar RNAs (snoRNAs), long non-coding RNAs (lncRNAs), and small non-coding RNAs (sncRNAs). With the advancements in next-generation sequencing technologies, extensive research has been performed to explore the roles of various sncRNAs (reviewed in Fu et al.^1^ and Madhry et al.^2^). Numerous studies related to their functions refute the previously marked statement about these sncRNA molecules, which were merely considered as ‘transcriptional junk.”^3^^,^^4^

There exist several kinds of sncRNAs, including microRNAs (miRNAs), small interfering RNAs (siRNAs), and piwiRNAs, among others. sncRNAs derived from tRNAs, called tRNA-derived small RNAs (tsRNAs), comprise tRNA-derived RNA fragments (tRFs) and tRNA halves (tiRNAs) and are an emerging class that has recently come into consideration. These tsRNAs are 14–40 nt in length, which originates from both precursor tRNA as well as mature tRNA by the action of different ribonucleases (RNases), including DICER, ELAC2, angiogenin (ANG), and RNase P, among others. These are predominantly generated during various kind of stress conditions.^5^^,^^6^ These tsRNAs are not just random byproducts, rather they have been found to be associated with several diseases and play various regulatory roles. This review discusses the biogenesis of tsRNAs and their functional roles in the context of various pathophysiological conditions, such as cancer, infectious diseases, and neurodegenerative diseases. This will help us to explore the potential of tsRNAs as disease-associated biomarkers and might also give us an insight for developing new therapeutic strategies.

## Biogenesis of tRNAs

**Figure 1:**
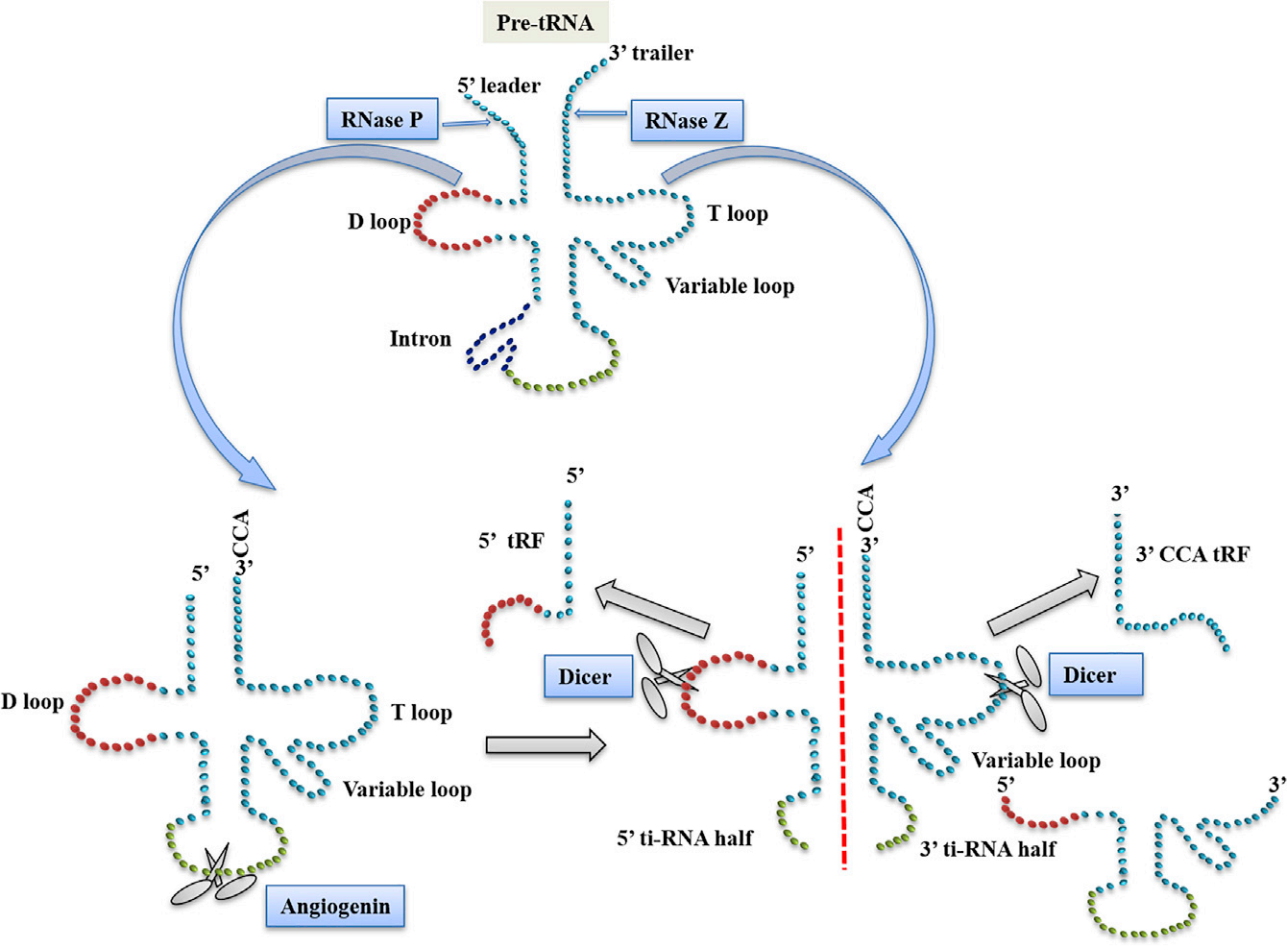
Schematic diagram representing processing of tRNA by various endonucleases for maturation and generation of tRNA-derived fragments Precursor tRNA is processed by RNase P and RNase Z at the 5′ end and 3′ end, respectively. tRNA intron is processed by the TSEN complex (not shown). Cleavage mediated by angiogenin and Dicer leads to generation of tiRNA halves and tRFs as shown in the diagram.

The human genome contains more than 500 tRNA-encoding genes, although a significant number among them are either not expressed or are expressed in a highly regulated cellular- or tissue-specific manner.^7^ Transcription of functional tRNA genes by RNA polymerase III (Pol III) generates precursor (pre-)tRNAs, which undergo further processing. This includes trimming of leader and trailer sequences, the addition of CCA residues at the 3′ end, and base modifications. This leads to the production of mature tRNAs that are exported to the cytosol by dedicated transporters. It has been proposed that different steps in this processing potentially happen in different loci within the nucleus.^8^ In eukaryotes, pre-mature tRNAs are recognized by the La protein, a highly abundant nuclear phosphoprotein that binds at the 3′ end and protects it from digestion by exonucleases.^9^^,^^10^ pre-tRNA molecules undergo certain stages of maturation before becoming translation-proficient tRNAs.^11^ Endonucleases such as RNase Z and RNase P trim the trailer and leader sequences, followed by intron splicing from their respective ends,^12^^,^^13^ as depicted in Figure 1. tRNA biogenesis sometimes may produce dysfunctional molecules. As a mechanism of quality control, they can be degraded either in the nucleus or in the cytosol by various methods.^9^^,^^14^

## tRNA halves and tRNA fragments

The first information of tRNA cleavage was noticed in *E. coli*, generated in response to bacteriophage infection.^15^ Zhao et al.^16^ have demonstrated the functional role of tRNA fragments purified from human urinary bladder carcinoma. Purified tRFs were 10–16 nt in length and these tRFs were competent to inhibit the growth of endothelial cells *in vitro*.^16^ Two studies carried out simultaneously in 2009 reported the generation of ANG-induced tRNA fragments from human fetus hepatic tissue^17^ and osteocarcinoma cells.^18^ Although initially considered as products of either random cleavage or cellular mechanism to degrade misprocessed tRNAs, tRFs that are often less than 40 nt in length are now known to be generated by precise ribonuclease activity in response to particular types of cell stress. In response to stress stimuli, unique phosphodiester bonds in the anti-codon loop of mature cytosolic tRNAs is cleaved by a specific ribonuclease, namely Rny1 in yeasts and ANG in mammals.^19^ This creates a break in the phosphodiester backbone of the tRNA and produces tRNA halves, which vary in length from 31 to 40 nt and are termed as 5′-tRNA halves (starting from the 5′ end of mature tRNA to the cleavage site in the anticodon loop) and 3′-tRNA halves (containing the sequence from the cleavage site in the anticodon loop to the 3′ end of the mature tRNA).^19^ In addition to this, next-generation sequencing studies have shown the existence of smaller stable tRFs, varying in length from 14 to 30 nt, which are termed as either tRF-5s, tRF-3s, or tRF-1s. tRF-5s and tRF-3s are derived from the extreme 5′ or 3′ end of mature tRNA through sequential endonucleolytic and exonucleolytic activity. tRF-1s corresponds to the trailer region of primary tRNA.^20^^,^^21^ tRF-5s is produced by endonuclease cleavage around the D-loop, while cleavage around the TψC loop results in generation of tRF-3s.^22^ Generation of the “mid-portion of 5′ end of specific tRNA” has also been reported where endonuclease cleavage happens at the anticodon loop and generates an ∼28- to 32-nt fragment.^23^

**Figure 2:**
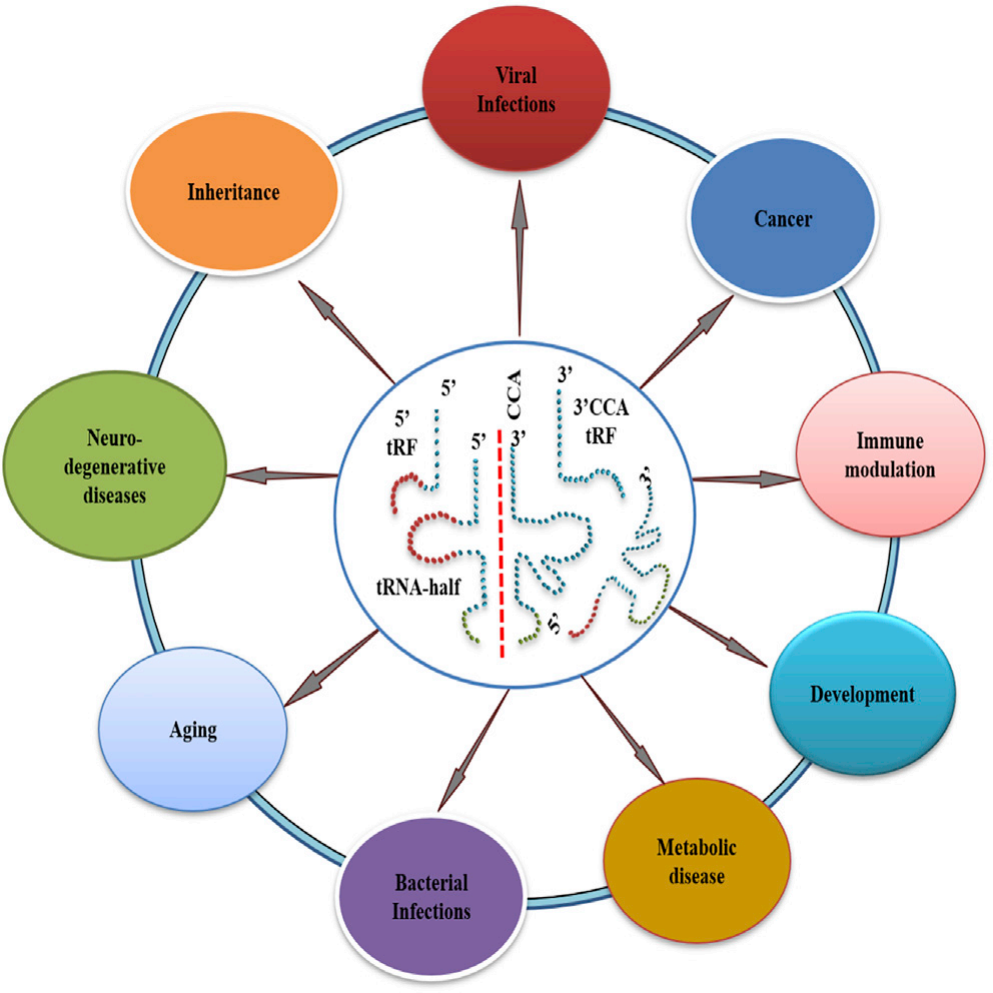
Schematic representation of roles of tRNA-derived RNA fragments in various human pathophysiologies

ANG-mediated tRNA cleavage commonly occurs at the anticodon loop of mature tRNA, leading to the accumulation of 5′- and 3′-tiRNAs in response to stress.^17^^,^^24^^,^^25^ Other mechanisms for tRNA cleavage have also been shown and reviewed elsewhere.^26^ Furthermore, it is not clear yet whether the two tRNA halves produced by cleavage of an individual tRNA molecule remain associated tightly or whether the cleavage encourages “bubbling” originating from the “broken” loop. Since the anti-codon loop of tRNA plays a crucial role in tRNA recognition by the aminoacyl tRNA synthetases, it is still not clear whether tRNA molecules with a break in the phosphodiester bond of the anti-codon loop are bound by the enzyme and/or charged with the cognate amino acid. Recently, Gonskikh et al.^27^ showed that the ribosome-bound 5′-tRNA half originating from proline tRNA is functionally capable of fine-tuning protein biosynthesis in a stress-dependent manner. They performed the screening of ribosome-bound tRFs in various mammalian cell lines and uncovered the mystery of the highly conserved tRNA^Pro^ 5ʹ half. They demonstrated that upon ribosome binding, the tRNA^Pro^ 5ʹ half inhibits protein biosynthesis with the concomitant appearance of a low-molecular-weight translation product. Functionally, tRNA halves (5′-tiRNAs and 3′-tiRNAs) and tRFs (i.e., tRF-5s, tRF-3s, or tRF-1s) seem to follow different mechanisms for their biological activity. Indeed, many investigations have indicated that only a fraction of cytosolic tRNAs is cleaved into tRNA halves during the stress response, and the levels of adaptor tRNA do not reduce critically enough to stop protein synthesis.^24^^,^^28^ tRFs are also produced by the activity of Dicer, a ribonuclease crucial for generation of mature miRNAs from pre-miRNAs.^29^ In addition to this, tRFs are known to associate with other components of the RNA-induced silencing complex (RISC), including multiple Argonaute proteins, indicating their potential involvement in miRNA/siRNA-like post-transcriptional gene silencing activity.^29^^,^^30^ Indeed, physiological mechanisms involving tRFs appear to be more diverse and complex when compared to other sncRNA pathways as shown in Figure 2. Many studies have suggested the functional relevance of tRNA fragments in the regulation of protein synthesis and cell proliferation.^31^^,^^32^ tRFs have also been shown to inhibit retrotransposition of long terminal repeat (LTR) retrotransposons or endogenous retroviruses (ERVs).^33^

Recent studies by various groups have indicated diversified functional roles for tRFs as regulatory factors in human pathophysiological conditions, including virus infections (Table 1).^5^^,^^6^^,^^65^^,^^66^ The mechanisms by which tRFs affect specific cellular processes in various pathological states are still largely unknown. Below, we discuss the tRNA fragments and their distinct regulatory roles in various human pathological states such as cancer, neurodegenerative diseases, metabolic diseases, various infectious diseases, and immunity as outlined in Figure 2.

**Table 1:** Functions of various tRNA-derived RNA fragments in human diseases

Types of disease	Types of tRFs, tsRNAs, and tiRNAs	Parent tRNA	Functions of tRFs, tsRNAs and tiRNAs	References
Cancer	3′-tRF-1001	tRNA-Ser^TGA^	promote prostate cancer cell proliferation	^34^
	tRF-544	tRNA-Phe^GAA^	significantly downregulated and associated with aggressive tumor growth	^5^^,^^6^
	tRF-315	tRNA-Lys^CTT^		
	3′-CCA-tRF (CU1276)	tRNA-Gly	gene silencing and post-transcriptional regulation in lymphoma cell lines	^29^
	tRF-19	tRNA-Arg^ACG^	differentially expressed in mammary epithelium and malignant breast tumors in response to deregulation of RUNX1	^35^
	tRF-29	tRNA-Tyr^GTA^		
	tRF-46	tRNA-His^GTG^		
	tRF-112	tRNA-Ser^GCT^		
	tRF-Glu	tRNA-Glu^YTC^	these tRFs interact with YBX1 protein and displace multiple oncogenic transcripts, which results in their instability and finally the suppression of breast cancer	^36^
	tRF-Asp	tRNA-Asp^GTC^		
	tRF-Gly	tRNA-Gly^TCC^		
	ts-4521	3′ ends of pre-tRNAs of serine and 3′ ends of pre-tRNAs of threonine	these tRFs interacts with Ago and Piwi-like proteins, which supports cancer cell proliferation in lungs	^37^^,^^38^
	ts-3676			
	tRF AS-tDR-000064	tRNA-Leu^AAG^	displayed abundant differential expression in pancreatic cancer cells compared to adjacent normal tissues	^39^
	tRF AS-tDR-000069	tRNA-Gln^CTG^		
	tRF AS-tDR-000102	tRNA-Ala^CGC^		
	tRF AS-tDR-001391	tRNA-Pro^CGG^		
	tRF AS-tDR-000064	tRNA-Leu^AAG^	qPCR data validated their upregulation in pancreatic cancer	^39^
	tRF AS-tDR-000069	tRNA-Gln^CTG^		
	tRF AS-tDR-000102	tRNA-Ala^CGC^		
	AS-tDR-001391	tRNA-Pro^CGG^	qPCR data validated its downregulation in pancreatic cancer	^39^
	5′-tRF-His	tRNA-His	inhibits the proliferation of breast cancer by regulating CKAP2	^40^
	tRF-Glu-CTC-003	tRNA-Glu^CTC^	functions are not characterized; however, these can be used as a biomarker in early breast cancer detection	^41^
	tRF-Gly-CCC-007 tRF-Gly-CCC-008 tRF-Leu-CAA-003	tRNA-Gly^CCC^		
	tRF-Ser-TGA-001	tRNA-Leu^CAA^		
	tRF-Ser-TGA-002	tRNA-Ser^TGA^		
		tRNA-Ser^TGA^		
	5′-tRNA-Val-CAC-2-1 half	tRNA-Val^CAC^	negatively regulates G_1_/S transition of mitotic cell cycle	^42^^,^^43^
	3′-tsRNA-CAG^Leu^	tRNA-Leu^CAG^	this 3′-tsRNA interacts with mRNA of ribosomal proteins RPS28 and RPS15, which results in translation promotion and finally leads to proliferation of hepatocellular carcinoma cells	^44^
	tRF-3017A	tRNA-Val ^TAC^	tRF-3017A regulates the NELL2 tumor suppressor gene and thus promotes metastasis in gastric cancer	^45^
Infectious disease	tRNA-Glu^CTC^	tRNA^Glu^	represses the target mRNA in the cytoplasm and facilitates RSV replication	^46^^,^^47^
	tRF5-Gly^CCC^	tRNA-Gly^CCC^	promote RSV replication	^46^
	tRF5-Lys^CTT^	tRNA-Lys^CTT^		
	tRF5-Glu^CTC^	tRNA^Glu^	suppresses APOER2 mRNA and facilitates RSV replication	^47^
	tRFs		binds with reverse transcriptase of human T cell leukemia virus-1 and alters replication	^48^
	tRF-3019	tRNA^Pro^		
	tRNA^Thr^ 3′ half (this tRF is produced in human pathogenic protozoa, *Trypanosoma brucei*)	tRNA^Thr^	binds with ribosome and stimulates protein biosynthesis during the stress recovery phase	^49^
	5′ and 3′ half of tRNAs (these tRFs are produced in human pathogenic fungus)	produced by endonucleolytic cleavage within the anticodon loop during conidiogenesis in *Aspergillus fumigatus*	protein synthesis is downregulated	^50^
	5′-tsRNA^Asp^	tRNA^Asp^	these tsRNAs interact with macrophages and support *Leishmania donovani* and *Leishmania braziliensis* to seek entry into cell cytoplasm	^23^
	5′-tsRNA^Gln^	tRNA^Gln^		
	3′-tRF-Ala^UGC^ (includes 3′-CCA)	tRNA-Ala^UGC^	this tRF stimulates TLRs and thus Thp1- and CTL1-mediated immune responses	^51^
	3′-tRF-Ser^TGA^	tRNA-Ser^TGA^	this 3′-tRF interacts with La/SSB proteins and resists IRES-mediated translation during HCV infection	^52^
Neurodegenerative diseases	5′-tiRNA-Ala and 5′-tiRNA-Cys	tRNA^Ala^ and tRNA^Cys^	these interact with YB-1 protein and induce the assembly of stress granules (SGs), which further generates a neuroprotective response	^53^
	5′-tiRNA^Asp^, tiRNA^Glu^, tiRNA^Gly^, tiRNA^His^, tiRNA^Lys^, tiRNA^Val^	tRNA^Asp^, tRNA^Glu^, tRNA^Gly^, tRNA^His^, tRNA^Lys^, tRNA^Val^	these 5′-tRNA halves accumulate as a result of Nsun2 and Dnmt2 methyltransferase mutation, which results in reduced translation and action of apoptotic and stress pathways; this further leads to reduced cell size of hippocampal, cortical. and striatal neurons	^54^
	3′- and 5′-tRNA halves	derived from different isoacceptor tRNAs such as tyrosine, leucine, and isoleucine	CLP1 protein deficiency in the case of pontocerebellar hypoplasia (PCH) results in unspliced pre-tRNA, which results in accumulation of 5′- and 3′-tRNA halves in the cells	^17^^,^^55^^,^^56^
Development and inheritance	5′-tRNA halves	derived from tRNA-Gly^GCC^, tRNA-Val^CAC^, tRNA-Gln^CTG^, tRNA-Glu^TTC^, tRNA-Lys^TTT^	these interact with IGF2BP1 and regulate the translation of cMyc, which further regulates cell proliferation and metabolism	^42^
	5′-tRFGly^GCC^	tRNA-Gly^GCC^	this targets MERVL an endogenous retroelement, which affects placentation and finally culminates in altered development of the offspring	^57^
Metabolic disease	tRF-3001b	tRNA-Asn^GTT^	this interacts with Prkaa1 and inhibits autophagy, which stimulates NAFLD development	^58^
	Gly-tRFs	tRNA^Gly^	involved in the progression of liver steatosis	^59^
Immunity	tRF5-Ala^CGC^	tRNA-Ala^CGC^	tRF5-Ala^CGC^ induces IL-8 production by stimulating p65	^60^
	5′-tiRNA-Glu^CTC^	tRNA-Glu^CTC^	this tRF induces chemokine and cytokine production	^47^
	tRF-Lys^AAA^	tRNA-Lys^AAA^	involved in immunity regulation	^61^
	tRF-Asp^GAY^	tRNA-Asp^GAY^		
Aging	tRF-3003a	tRNA-Cys^GCA^	regulates OA pathogenesis by repressing the Janus kinase 3 (JAK3) pathway	^62^
	tiRNA-5035-Glu^CTC^	both derived from tRNA-Glu^CTC^	differential expression studies showed the downregulation of these tRFs in metacarpophalangeal joints of old horses	^63^
	tiRNA-5031-Glu^CTC^-1			
	5′-tRNA halves	derived from tRNA-Asn^GTT^, tRNA-Ile^AAT^, and tRNA-Asp^GTC^	downregulation of these tRFs was observed in rheumatoid arthritis patients, which suggests its use as a putative biomarker	^64^

## tsRNAs in infectious diseases

Various studies have shown that many pathogens secrete exosomes that contain different ncRNA-derived along with tRNA-derived fragments that are known to induce interleukins (ILs) and interferon (IFN) production.^67^ Lambertz et al.^23^ conducted their study on protozoan parasites *Leishmania donovani* and *Leishmania braziliensis* and reported that parasites secrete exosomes containing mRNA and sncRNA cargos. These RNAs were capable of modulating recipient cell phenotypes to support chronic infection. These exosomes deliver their ncRNA cargos to the macrophages, which support host-parasite interactions for seeking entry into the cell cytoplasm. RNA sequencing (RNA-seq) analysis of exosomal RNAs indicated that most of the sequences were derived from ncRNA species. Further in-depth analysis reveals the presence of tRFs with regulatory functions. Identified tRNA fragments were derived from the 5′ end or mid-5′ end (anticodon loop) and the 3′ end of specific tRNAs. Those identified tRFs and tiRNAs are tRNA^Asp^, tRNA^Gln^, tRNA^Glu^, and tRNA^Leu^, in which tsRNA^Asp^ and tsRNA^Gln^ were found to be most abundant.^23^ Similar studies were reported with *Trypanosoma cruzi* that show that extracellular vesicles released by *T. cruzi* contained tRNA-derived halves along with other ncRNAs that are crucial for promoting its replication cycles and to confer infection in the susceptible cells.^68^ Various studies have also been carried out in the context of *Mycobacterium* and tRNA or its derived fragments. Obregón-Henao et al.^69^ have shown that tRNA and rRNA fragments induces early apoptosis in host monocytes by modulating a caspase-8-dependent pathway that attenuates host immune response toward mycobacterium.^69^ Further studies have reported that tRNA fragments can activate surface pattern-recognition receptors (PRRs) such as Toll-like receptors (TLRs), which play a pivotal role in the mammalian host immune response.^70^ These receptors are either expressed extracellularly on the cell surface or intracellularly in response to the infection, which are sensed by the immune cells and lead to the production of IFN glycoproteins.^71^ Various studies have shown that tRNA-derived fragments are capable of inducing immunity probably through activation of TLRs. Fragments of tRNA-Ala^UGC^ that include the 3′ terminal CCA were shown to function as efficient adjuvants stimulating TLR3 and inducing the generation of T helper 1 (Th1) and cytotoxic T lymphocytes (CTLs) against a co-administered subunit vaccine antigen.^51^ In addition to TLR3, Pawar et al.^72^ have unveiled the role of 5′-tRNA-His^GUG^ half in activating the TLR7 which is produced in response to infection by *Mycobacterium tuberculosis*.^72^

Different studies have also discussed the tRFs 5′- and 3′-tiRNA generation and their functional role during viral infections. Generation of tRFs and their related roles are very well studied in respiratory syncytial virus (RSV) infection. Wang et al.^46^ have shown that RSV infection leads to the induction of a functional 5′-tiRNA-Glu^CTC^. They further demonstrated that production of cytosolic 5′-tiRNA-Glu^CTC^ is independent of Dicer or Drosha and specifically produced in an ANG-dependent manner upon RSV infection. These 5′-tiRNAs enhance viral replication by inhibiting the translation of APOER2 by interacting with the 3′ end of the cognate mRNA.^46^^,^^47^ Further studies conducted by Zhou et al.^73^ have examined the role of three RSV-induced tiRNAs (tRF5-Gly^CCC^, tRF5-Lys^CTT^, and tRF5-Cys^GCA^) derived from the 5′ end of mature tRNAs. They found that, out of these three 5′-tRFs, tRF5-Gly^CCC^ and tRF5-Lys^CTT^ were functionally competent in inducing the production of pro-inflammatory cytokines such as IL-6, IL-8, and RANTES.^73^ Selitsky et al.^74^ used high-throughput sequencing to profile small RNAs (14–40 nt) in the liver from Japanese patients. These patients were suffering from advanced hepatitis B virus (HBV) and hepatitis C virus (HCV) and hepatocellular carcinoma (HCC). They reported the abundance of 30- to 35-nt-long 5′ tiRNA in the non-malignant liver with a compelling increase in humans and chimpanzees with chronic viral hepatitis. They also correlated ANG expression levels with 5′-tiRNA generation.^74^ In a similar study, a tRF3 derived from tRNA^pro^ (tRF-3019) was shown to be capable of priming reverse transcription of human T cell leukemia virus-1 (HTLV1) genome and in fact be packaged into viral particles.^48^ Recently, it has also been observed that 3′-derived tRFs possess complementarity with the primer-binding site (PBS) predominantly found in the LTR retroelements that utilize the 3′ end of tRNA for reverse transcription. It has been found that 18-nt tRF (tRF3a) specifically targets reverse transcription whereas 22-nt tRF (tRF3b) silences transposons of LTR retrotransposons or endogenous retroviruses (ERVs).^33^ Cho et al.^52^ have reported that the pre-tRNA-Ser^TGA^-derived 3′-tRF trailer interacts with La/SSB protein, which acts as a RNA chaperon to provide stability to the nascent transcripts produced by RNA Pol III. La/SSB also plays a significant role in HCV internal ribosome entry site (IRES)-mediated translation. The interaction between tRF and La/SSB leads to limited availability of cytoplasmic La/SSB, which inhibits HCV IRES-mediated translation.^52^

These studies suggest that tRFs play a significant role in host-pathogen interactions. They regulate host-pathogen interaction by various mechanisms such as gene targeting at the posttranscriptional level, intercellular and intracellular communications, and immune regulations during infectious diseases. Further characterization of tRFs function in infectious diseases will thus answer the possibility of therapeutic applications of these tRFs.

## tsRNAs and cancer

Cancer is a multifactorial disease characterized by uncontrolled proliferation of cells and their invasion into either surrounding tissues or metastasis to distant sites within the organism. Deregulation of cell proliferation mostly results from defects in signaling pathways and/or gene expression patterns.^75^^,^^76^

tRFs are known to regulate protein translation by interacting with ribosomes and aminoacyl tRNA synthetases.^77^^,^^78^ They also potentially interact with RNA-binding proteins, ribosomal proteins, Ago proteins, and Piwi-interacting proteins for regulating gene expression in a cell type-specific manner,^44^^,^^79^ which suggests their possible roles in tumorigenesis. Goodarzi et al.^36^ found that under stressed conditions, cancer cells produce specific tRFs derived from tRNA^Asp^, tRNA^Glu^, tRNA^Tyr^, and tRNA^Gly^, which interact with YBX1 protein. YBX1 (Y-box binding protein 1) is known to bind oncogene transcripts in breast cancer and enhance their stability, which further leads to an increase of cell proliferation and promotes cancer. Contrarily, tRFs produced during cancer compete with the oncogene transcripts to bind with YBX1 and thus suppress cancer cell growth.^36^ Kim et al.^44^ showed that specific tRNA fragments such as tsRNA-Leu^CAG^, a 3′-tsRNA, functionally interact with mRNA of ribosomal proteins RPS28 and RPS15. This interaction results in enhanced translation, which leads to the proliferation of hepatocellular carcinoma cells.^44^ Some tRFs interact with Ago and piwi proteins, suggesting their miRNA- or piwi-interacting RNA (piRNA)-like functions.^80^^,^^81^ In lung cancer it has been observed that tRNA-Ser- and tRNA-Thr-derived ts-4521 and ts-3676, respectively, interact with Ago1 and Ago2 along with piwi-like protein 2 (Piwil2).^37^^,^^38^ Downregulation of tRNA^Ser^-derived ts-4521 leads to cell proliferation.^82^

tRNA fragments in cancer can also be produced as a result of induction of specific endonucleases. 3′-tRFs (e.g., tRF-1001) are produced by the cleavage of the cytoplasmic pre-tRNA-Ser^TGA^ by ELAC2. Overexpression of this tRF was seen in different cancer cell lines with specific correlation with proliferation of prostate cancer cells.^34^ In addition, the expression of ANG has been shown to be significantly increased in breast cancer tissue, which was also marked as a favorable prognostic marker in primary breast carcinoma.^83^ Furthermore, ANG-induced tiRNAs have been demonstrated to inhibit apoptosis through interaction with cytochrome *c* and directly contribute to ANG-mediated angiogenesis and cancer cell proliferation.^84^

A direct correlation of tRNA fragments with metastasis and cell proliferation has been shown recently. Tong et al.^45^ have reported that tRNA-Val^TAC^-derived tRF-3017A promotes metastasis in gastric cancer by regulating the NELL2 tumor suppressor gene.^45^ The tumor suppressor transcription factor (TF) RUNX1 plays a significant role in initiation and metastasis of breast cancer. Interestingly, RUNX1 has been demonstrated to suppress the expression of four tsRNAs, i.e., ts-19, ts-29, ts-46, and ts-112.^35^^,^^85^ Furthermore, intracellular levels of ts-112 showed a positive correlation with the proliferation rate of the breast tissue epithelial cell line MCF10A.^35^ Mohammed et al.^86^ reported the polymorphic mutation of mitochondrial gene A12308G in tumor samples from colorectal cancer patients. This mutation leads to the alteration of secondary and tertiary structure of tRNA-Leu^CUN^, which was considered as a valuable molecular target in colorectal cancer.^86^ On the contrary, 22-nt-long 3′-tRF derived from tRF-Leu^CAG^ is shown to promote cancerous cell survival in lung cancer.^87^

Since the prognosis of cancer improves dramatically with an earlier diagnosis, a lot of attention is being given to the discovery of early biomarkers.^88^ The presence of tsRNA has been found to be associated with particular types of cancer, for exmaple, 5′-tRNA-Val^CAC^-2-1 with oral squamous cell carcinoma (OSCC), tiRNA-5034-Glu^TTC^-2 with gastric carcinoma, and 5′-tRNA-Glu^CUC^ in prostate cancer.^89^^,^^90^ In studies performed to explore the possible use of tRFs as a diagnostic biomarker for early stage detection of breast cancer, out of 30 selected tRFs, 6 (tRF-Glu^CTC^-003, tRF-Gly^CCC^-007, tRF-Gly^CCC^-008, tRF-Leu^CAA^-003, tRF-Ser^TGA^-001, and tRF-Ser^TGA^-002) derived from the 5′ end of five different tRNAs were shown to have significantly decreased expression in cancerous tissue when compared to control samples.^41^ Hence, these tRFs could potentially be used as a diagnostic biomarker to detect and follow the progression of breast cancer. A study comparing cancerous pancreatic cells and adjacent normal tissue showed differential regulation of 48 tRFs.^39^ tRFs AS-tDR-000064, AS-tDR-000069, AS-tDR-000102, and AS-tDR001391 exhibited abundant differential expression in pancreatic cancer cells compared to adjacent normal tissue samples. Investigations into the global expression profile of small RNA (small RNome) in prostate cancer cells highlighted the abundance of tsRNAs.^39^ Most of the identified tRFs are derived from the 5′ and 3′ ends of mature cytosolic tRNAs. It has also been shown that tRFs can be generated from pre-tRNAs, leader sequences, and from mitochondrial tRNAs.^91^ Such tRFs have been further classified as (1) 5e-tRFs with a start position in the first nucleotide of the 5′ end of the tRNA (“e” stands for “end”); (2) D-tRFs with a start position between nucleotides 12 and 23 and overlapping the D-loop of the pre-tRNA; and (3) A-tRFs starting between nucleotides 31 and 39 and overlapping with the anticodon loop.^91^ Olvedy et al.^91^ evaluated expression of the tRFs pool via RNA-seq from fresh-frozen patient samples derived from normal adjacent prostate and different stages of prostate cancer and found that the 5′-e-tRFs are abundantly expressed in prostate cancer cells.^91^ The differential expression of these 5′-e-tRFs among normal adjacent prostate and prostate cancer was associated with malignant transformation. Alternatively, 3′-e-tRFs are downregulated in the prostate cancer cells. Comparison of various research data indicates that downregulated tRFs derived from the 3′ end of tRNA could be a key event in the progression of cancer.^29^^,^^34^ Differentially expressed tRNA fragments thus can be used as biomarkers to detect the level and progression of cancer.

## tsRNAs in neurodegenerative diseases

Defects in tRNA metabolism or associated processing enzymes often leads to various neurological disorders. ANG mutations are reported in patients suffering from different neurological disorders, suggesting a direct involvement in pathways that lead to motor and dopaminergic neuron degeneration. The modulated RNase activity of mutant ANG has shown its implications in the pathogenesis of amyotrophic lateral sclerosis (ALS) and Parkinson’s disease (PD).^92^^,^^93^ In ALS patients, mutated ANG leads to the generation of 5′-tiRNAs, which are derived from tRNA-Ala and tRNA-Cys. These tiRNAs interact with translational repressor protein YB-1 to inhibit translation and induce the assembly of stress granules (SGs). Furthermore, these tRNA halves assemble a G-quadruplex structure that mediates its entry into motor neurons to carry out the neuro-protective response. C9ORF72 is the leading genetic cause of ALS, which consists of GGGGCC repeats. These repeats also form a G-quadruplex, which inhibits the functions of tRNA-Ala halves to induce SG assembly. This functional interaction between G-quadruplex of tiRNAs and C9ORF72 results in ALS pathology.^53^

A study by Blanco et al.^54^ showed that mice and humans with mutated cytosine-5 RNA methyltransferase NSun2 and Dnmt2 develop microcephaly and other neurological abnormalities. They also showed that NSun2 and Dnmt2 deficiency induces ANG ribonucleases activity, which leads to the accumulation of 5′-tRFs. The accumulation of 5′-tRFs results from high-affinity binding between ANG and tRNA lacking NSun2-mediated methylation. These 5′-tRFs further result in reduced translation and action of apoptotic and stress pathways, which ultimately culminates in the reduced cell size of hippocampal, cortical, and striatal neurons.^54^

In pontocerebellar hypoplasia (PCH), *CLP1* mutation was reported. CLP1 is a kinase protein that is primarily associated with pre-tRNA processing and generates mature tRNAs. Mutated CLP1 results in accumulation of unspliced pre-tRNAs and depleted mature tRNA levels of different isoacceptor tRNAs such as tyrosine, leucine, and isoleucine. Due to mutated CLP1 protein, spliced tRNA could not get ligated, which results in accumulation of tiRNAs, which leads to reduced protein translation and cell death.^17^^,^^55^^,^^56^

## tsRNAs in development

Stem cells carry the potential for extensive division and differentiation, which helps in embryonic development and tissue regeneration. Particularly, mouse embryonic stem cells (mESCs) have the capacity for infinite self-renewal and differentiation into any cell type.^94^ In a recent study carried out by Krishna et al.^42^ it was observed that mESCs abundantly consists of 5′-tiRNAs as compared to miRNAs. They demonstrated the increased expression of specific 5′-tiRNAs in a retinoic acid (RA)-induced mESC differentiated mouse model. These tiRNAs were specifically derived from certain tRNAs such as tRNA-Gly^GCC^, tRNA-Val^CAC^, tRNA-Gln^CTG^, tRNA-Glu^TTC^, and tRNA-Lys^TTT.^ Functional studies of these tRFs revealed that they inhibit pluripotent genes and lead cell differentiation in a lineage-specific manner.^42^ Furthermore, RNA immunoprecipitation studies led to the identification of IGF2BP1, which interacts with these tiRNAs and regulates the translation of cMyc, which is known as the “master regulator” of cell proliferation and metabolism.^42^^,^^43^ ESCs are also known to express tRFS-5s (18 nt long). These are derived from the T-loop of certain tRNAs based on pseudouridine modification. The PUS7 enzyme is involved in uridine modification. This is the key enzyme in processing tRF formation. Downregulation of this enzyme leads to decreased expression of tRFS-5s, which is attributed to the dysregulated formation of the germ layer. In hematopoietic stem cells, downregulated PUS7 leads to the upregulation of 5′-tiRNAs.^95^ Using deep sequencing data analysis, Su et al.^96^ have reported differential expression of tRFs in placenta/decidua, fetal brain, and fetal liver.^96^ Furthermore, the expression of tRFs changes dynamically as embryonic development progress. Maternal immune activation by injecting viral infection mimetic in pregnant mice leads to sudden changes in the expression levels of specific tRFs.^96^

## tsRNAs in metabolic disease

Accumulating evidence has suggested that biogenesis of tRFs occurs in response to various stress conditions.^17^^,^^18^^,^^29^^,^^97^ Non-alcoholic fatty liver disease (NAFLD) is a metabolic disorder defined by fat deposition within the liver that leads to liver fibrosis. Oxidative stress and metabolic deregulation are the major factors responsible for the progression of NAFLD.^98^^,^^99^ To study the role of tRFs in NAFLD, Zhu et al.^58^ analyzed the RNA transcriptome profile of both NAFLD liver tissues and control mice.^58^ They observed a total of nine upregulated tRFs in NAFLD liver compared to normal liver. Further Gene Ontology (GO) and target gene prediction studies revealed five among nine tRFs that could potentially target genes involved in autophagy-related pathways such as the Wnt signaling pathway, the mTOR signaling pathway, and the phosphatidylinositol 3-kinase (PI3K)-Akt signaling pathway. Quantitative PCR results confirm that tRF-3001b was significantly overexpressed compared to the other four tRFs. Finally, functional assays confirm that tRF-3001b targets and inhibits Prkaa1 expression, an autophagy-related gene. Inhibition of Praka1, in turn, leads to the impairment of autophagy and aggravates the development of NAFLD.^58^ Zhong et al.^59^ investigated the role of tRFs in AFLD, and their preliminary experiments revealed that tRF-Gly is involved in the progression of liver steatosis.^59^ Furthermore, they also demonstrated that tRF-Gly is overexpressed in mice fed with ethanol, and inhibition of tRF-Gly decreased alcohol-induced hepatosteatosis. Knockdown and knockout experiments of complement proteins by Zhong et al.^59^ revealed that deficiency of C3 represses the production of tRF-Gly, which was unaffected in C5 deficiency.^59^ Hepatic tissue transcriptome profiling concludes that sirtuin1 (Sirt1) expression was upregulated upon inhibition of tRF-Gly. Expression of Sirt1 regulates lipid metabolism, including lipogenesis and β-oxidation pathways, and hence it plays a pivotal role in AFLD progression.^100^^,^^101^

## tsRNAs in inheritance

Increasing lines of evidence from animal studies and human famines suggest that parental environmental exposure can affect the germline and influence their progeny through epigenetic mechanisms.^102^^,^^103^ Chen et al.^104^ and Sharma et al.^57^ simultaneously carried out seminal studies that mark the importance of tRNA fragments during transgenerational inheritance. Chen et al. showed that a high-fat diet (HFD) regulates tRF expression, which was finally attributed to the altered metabolic phenotype in the offspring. A HFD (with 60% fat) or normal diet (ND with 10% fat) was fed to the paternal mice. Offspring generated from the sperm of HFD male mice showed impaired glucose tolerance and insulin resistance at the age of 7 weeks. To understand the mechanism, the authors analyzed RNA modification profiles of HFD and ND sperm tsRNA. Interestingly, two RNA modifications, namely 5-methylcytidine (m^5^C) and *N*^2^-methylguanosine (m^2^G), were found to be significantly upregulated in HFD group sperm.^104^ Another similar study carried out by Sharma et al.^57^ has shed light on small RNA biogenesis in the context of the effect of low protein dietary regulation during post-testicular sperm maturation. Sharma et al. demonstrated that the paternal diet can affect offspring metabolism via information located in sperm. They carried out their study on male mice consuming a low-protein diet and used their sperm to perform the *in vitro* fertilization (IVF) and tested the altered expression of metabolic genes. They found that IVF-derived offspring of male mice (fed on a low-protein diet) exhibited significant hepatic upregulation of the certain gene encoding the cholesterol biosynthesis enzyme squalene epoxidase in comparison to control IVF offspring. They suggest that tRF-Gly^GCC^ is regulated by a low-protein diet and it targets a MERVL endogenous retroelement. tRF-Gly^GCC^-mediated repression of MERVL-regulated genes may affect placentation and finally culminate in altered development of the offspring.^57^ tRF-Gly^GCC^ has been shown to have the characteristic of dimerization. This property protects tRFs against the action of cellular nucleases.^105^ These results suggest that tRFs possess intergenerational attributes. Hua et al.^106^ investigated differential expression of sncRNAs to evaluate the quality of sperm during IVF. Their results suggest that tsRNAs along with rRNA-derived small RNAs (rsRNAs) may be potential clinical biomarkers to assess sperm quality during IVF.^104^^,^^106^ During maturation, sperm travels from caput to corpus and finally to the cauda region. tRF pools are found in cauda sperm, suggesting their functional role in zygote formation after fertilization.^106^^,^^107^

## tsRNAs in immunity

Recent studies have suggested the roles of tRNA fragments in eliciting an immune response. Dendritic cells (DCs), when interacting with T cells, release multi-vesicular bodies (MVBs) containing shuttle RNAs, including tRFs, which mediate communication between these cells to ensure the immune response.^108^^,^^109^ The expression of an abundant quantity of tRFs derived from 3′ and 5′ ends of tRNA was found in the cytoplasm of the immune cells, specifically DCs, using deep-sequencing studies. Further experimental results suggested that tRFs derived from tRNA-Lys^AAA^ were enriched in both cellular and cell-derived vesicles (shuttle RNA), whereas an enriched concentration of tRFs derived from tRNA-Asp^GAY^ was present in immune cell cytoplasm.^61^ In LPS-stimulated monocytes, various tRNA fragments were found, but primarily tRF-Tyr^GTA^ was found to be upregulated, whose function is yet to be discovered.^110^ Maute et al.^29^ demonstrated that CU1276, which is derived from 3′-tRFs, generated in a DICER-I-dependent manner interacts with Ago proteins to suppress mRNA targets. Surprisingly, this CU1276 is downregulated in a Burkett lymphoma cell line, which is a germinal center-derived cell line. Downregulated CU1276 affects cell proliferation and causes DNA damage by inhibiting RPA1 (replication protein A1) that plays a dynamic role in DNA metabolism, including replication, recombination, and DNA damage and repair.^29^

Gong et al.^25^ have reported that *Rickettsia* infection leads to ANG upregulation in mouse endothelial tissue. This upregulated ANG induces tRNA fragments such as tRNA-Val^GTG^ and tRNA-Gly^GCC^, which are probable mediators of the immune response and autophagy.^25^ Stress induced by arsenite also leads to the generation of tRFs, in which tRF5-Ala^CGC^ induces IL-8 production by stimulating transcription factor p65.^60^

Different viruses also generate tRFs, which mediate some type of immune response. During RSV infection, 5′ halves derived from tRNA-Glu^CTC^ induce chemokine and cytokine production.^47^ Bovine leukemia virus (BLV) generates fragments from tRNA-Gln^CTG^, tRNA-His^GTG^, and tRNA-Gln^TTG^, which induces an antibody response and thus can be categorized as potential biomarkers.^111^

Immune cells have pools of 3′- and 5′-tRFs in their cytoplasm, while only 5′-tRFs are enriched in immune cell-derived vesicles.^61^ tRFs, when used as an adjunct therapy along with HBV surface antigen, interact with TLR3 and elicit CTLl and Th1-mediated immunity.^51^ tRNA secreted by bacteria also interacts with TLR7, which leads to an IFN-mediated response.^112^ Other small RNAs, such as miRNAs, also stimulate cell-mediated and humoral immune responses by interacting with various TLRs.^113^

## tsRNAs in aging

Aging is a constitutive change in all parts of the body, including the brain, which commonly shrinks in volume, and is followed by large size of sulci.^114^ It is characterized by a progressive loss of physiological integrity, leading to improper functioning and increased vulnerability to the death of living organisms. This integration is the primary risk factor for major human pathophysiological diseases. Studies suggest that tRFs have been found to be associated with aging-related neurodegeneration. Senescence-accelerated mouse prone 8 (SAMP8) was used as a model to study tRF expression patterns during age-related neurodegeneration, and a senescence-accelerated mouse resistant 1 (SAMR1) model was used as a control. SAMP8 mice were used, as it is known that they exhibit age-related brain degeneration, for example, Alzheimer’s disease, Parkinson’s disease, and like pathologies. Sequencing analysis suggests that eight tRFs, namely, AS-tDR-011775, AS-tDR-006835, AS-tDR-012690, AS-tDR-013428, AS-tDR-005058, AS-tDR-011389, AS-tDR-010789, and AS- tDR-011670, were differentially expressed in a SAMP8 and SAMR1 mouse model. GO and Kyoto Encyclopedia of Genes and Genomes (KEGG) analysis showed that these tRFs were found to be involved in the regulation of such gene expression involved in brain-related functions, which are affected by aging.^115^ Similarly, studies on the expression pattern of tRFs in young, middle-aged, and old rats showed aged-dependent expression of tRFs. 3′-tRFs were found to be increased with age, and most of these tRFs were found to be interacting with genes such as netrin receptor, cadherin genes, and fibroblast growth factor receptor 2 genes, which have important roles in nervous system development. Thus, this result suggests that tRFs might be playing an important role in modulating gene expression in nervous system diseases depending upon age.^116^

Osteoarthritis (OA), rheumatoid arthritis (RA), and aging are independent processes; however, aging-related events lead to OA and RA. Studies have revealed that stress, a key hallmark of aging, promotes OA through cartilage degradation.^117^^,^^118^ Goldring^62^ investigated the altered expression of tRFs in chondrocyte cells upon IL-1β stimulation, which is an inflammatory cytokine and is known to perform a critical role in the development of OA pathogenesis chondrocyte cells.^62^^,^^63^ Results confirm that the IL-1β stimulates the overexpression of tRF-3003a, a tRF derived from tRNA-Cys^GCA^. Further analysis confirms that the overexpression of tRF-3003a leads to inhibition of JAK3 expression. Inhibition of JAK3 significantly reduces IL-6, which has been shown to be associated with OA pathogenesis and progression.^62^ A recent study by Balaskas et al.^63^ analyzed the sncRNAs, including tRNAs and their fragments, from the chondrocytes of healthy metacarpophalangeal joints of both young and old horses (horses have been used as a model of OA) and also compared these findings with tRNAs and their respective fragments of human OA cartilage.^63^ Extracted RNAs were subjected to RNA-seq, and differential expressions of a total of 83 sncRNAs (including miRNAs, snoRNAs, snRNAs, and tRNAs) were observed. Further tRNA fragment analysis suggested that tiRNA-5035-Glu^CTC^ and tiRNA-5031-Glu^CTC^-1 tiRNAs were downregulated in both high-grade OA human cartilage and old equine chondrocytes. The results showed that aged equine samples have changes in the expression of specific tRNAs and tRFs when compared to young equine samples and found the induction of tiRNA-Glu^CTC^ and tiRNA-His^GTG^ in old compared to young equine chondrocytes and in high-grade compared to low-grade cartilage. Along with these tiRNAs, the parent tRNA-Cys^GCA^ was also found to be increased in both aged equine chondrocytes and high-grade OA human cartilage samples.^63^ Similarly, Ormseth et al.^64^ constructed a snRNA library using RNA isolated from endogenous plasma of RA patients. tRFs produced from the 5′ end of tRNA-Asn^GTT^, tRNA-Ile^AAT^, and tRNA-Asp^GTC^ are downregulated. Intriguingly, increased concentrations of tRFs derived from naturally occurring suppressor tRNAs were noticed in the endogenous plasma of RA patients.^64^ These studies support a role for tRNA-derived RNA fragments in aging cartilage and their potential involvement in age-related diseases.

## Conclusions and future perspective

tRNA-derived RNA fragments are the new entrant in the field of small ncRNAs. These fragments are generated from different regions of a tRNA molecule. Their generation depends on personal attributes, cell and tissue specificity, environmental (stress) conditions, and tRNA modifications. They have a functional role in various pathophysiologies.

Earlier these fragments were considered to be junk byproducts. Their specificity in several biological processes suggests that these fragments can regulate various cellular functions, including fine modulation of gene expression, through either transcriptional, post-transcriptional, or both mechanisms, in response to internal and external stress.

This review highlights some exciting aspects of fragmentation of tRNAs and their role in various pathophysiologies. Furthermore, we explore herein the possibilities to use tRNA fragments as biomarkers for the severity of disease or therapeutic potential. These tRNA fragments interact with TLRs and mediate the immune response. Wang et al.^51^ have highlighted the role of various tRNA fragments as an adjuvant to elicit strong Th1 and CTL immune responses. In the case of RSV, 5′-tRNA halves are generated from tRNA-Gly^GCC^ as a result of ANG induction. These tiRNAs promote virus replication and thus can potentially be targeted to control RSV infection. tRNA-derived fragment mimics or anti-sense oligonucleotides (ASOs) against ideal tRF candidates can be used to explore the therapeutic application of tRFs.

YBX1 is a protein that stabilizes oncogene transcripts and accelerates cell proliferation in breast cancer. Some tRNA fragments were found to interact with YBX1, which can suppress cell proliferation. Similarly, tRNA-Ser-derived ts-4521 also suppresses cell proliferation in lung cancer. These tRNA fragments can thus potentially be used as mimetic tRNA therapy against breast and lung cancer. In another case, a study on gastric cancer has shown that tRF-3017A promotes metastasis. tRF-Leu^CAG^ is also known to promote cancerous cell survival in lung cancer. ASOs can thus potentially be targeted against these tRNA fragments. ASOs can work as inhibitors to inhibit the roles of the tRNA fragments that support cancerous cell growth and metastasis. tRFs involved in aging-related processes such as neurodegeneration, OA, and RA can be used as biomarkers. Along with these highlighted roles of tRFs in human diseases, they also serve as conduits for the transfer of information across progeny. During early embryogenesis and inheritance, 5′-tiRNA pools have been shown to be localized in the cauda region of sperm, which might get transferred to oocytes during fertilization.

The field of tRNA-derived RNA fragments is still at its early stage. There are many unanswered questions related to their biogenesis and functional roles. Advancements in deep-sequencing technology are needed to overcome various technical challenges. First, the tRNA structure is highly prone to modifications, which makes it difficult for sequencing and further characterization. Second, it is hard to discriminate among the reads generated by pre-tRNA or mature tRNA. Third, tsRNA generation particularly depends on multiple variables such as tissues specificity, disease conditions, cellular stresses, and presence of various modifications in tRNAs, etc. These factors complicate the identification of tRNA-derived RNA fragments generated from isodecoder tRNAs, which share sequence similarity but have different chromosomal locations. Technical advancement and more comprehensive studies are needed to discover the potential of tRNA-derived fragments as putative biomarkers or prognostic markers and also to discover the strategies for prospective therapeutics either as a stand-alone therapy or as an adjunct therapy.
